# An Aerial Image Detection Algorithm Based on Improved YOLOv5

**DOI:** 10.3390/s24082619

**Published:** 2024-04-19

**Authors:** Dan Shan, Zhi Yang, Xiaofeng Wang, Xiangdong Meng, Guangwei Zhang

**Affiliations:** 1 School of Mechatronical Engineering, Beijing Institute of Technology, Beijing 100081, China; shandan0405@sjzu.edu.cn (D.S.); xfwang@bit.edu.cn (X.W.); 7520220037@bit.edu.cn (G.Z.); 2 School of Electrical and Control Engineering, Shenyang Jianzhu University, Shenyang 110168, China; mengxiangdong@stu.sjzu.edu.cn

**Keywords:** aerial images, YOLOv5, mixed attention module, BiFPN

## Abstract

To enhance aerial image detection in complex environments characterized by multiple small targets and mutual occlusion, we propose an aerial target detection algorithm based on an improved version of YOLOv5 in this paper. Firstly, we employ an improved Mosaic algorithm to address redundant boundaries arising from varying image scales and to augment the training sample size, thereby enhancing detection accuracy. Secondly, we integrate the constructed hybrid attention module into the backbone network to enhance the model’s capability in extracting pertinent feature information. Subsequently, we incorporate feature fusion layer 7 and P2 fusion into the neck network, leading to a notable enhancement in the model’s capability to detect small targets. Finally, we replace the original PAN + FPN network structure with the optimized BiFPN (Bidirectional Feature Pyramid Network) to enable the model to preserve deeper semantic information, thereby enhancing detection capabilities for dense objects. Experimental results indicate a substantial improvement in both the detection accuracy and speed of the enhanced algorithm compared to its original version. It is noteworthy that the enhanced algorithm exhibits a markedly improved detection performance for aerial images, particularly under real-time conditions.

## 1. Introduction

Owing to their advantageous characteristics, such as compact size, energy efficiency, minimal noise, and terrain adaptability, UAVs (unmanned aerial vehicles) are extensively utilized for image capture across diverse domains [1]. Nevertheless, the varying altitudes of UAV flight and shooting perspectives result in aerial images containing numerous small target objects, disordered object arrangements, and complex backgrounds, posing significant challenges to the task of target detection [2].

In recent years, the rapid advancement of deep learning has led to notable breakthroughs in the field of computer vision [3]. Concurrently, deep-learning-based target detection methods have emerged as the predominant approaches in the field, owing to their characteristics of requiring no manual feature extraction, robust adaptability, and high resilience. Presently, deep-learning-based target detection methods are primarily categorized into two groups: those based on the target detection framework and those focused on regression problems. Algorithms such as R-CNN [4], Fast RCNN [5], Faster RCNN [6], etc., belong to the former category. These algorithms extract feature information from candidate regions, followed by regression classification based on this information, resulting in a two-stage process. While this approach yields higher recognition accuracy, it comes at the cost of slower detection speed. The other category comprises algorithms focused on regression problems. Representative algorithms in this category include the YOLO [7] series and SDD [8], among others. These algorithms offer the advantages of faster detection speed and ease of deployment, attributed to the parallelization of regression and classification tasks.

Owing to variations in drone flight altitude and shooting angles, objects in aerial images display significant texture and shape discrepancies compared to objects in horizontally captured natural images. Zhang et al. [9] introduced a lightweight CNN-based method for UAV aerial image detection, focusing on real-time processing and detection accuracy. Nevertheless, the overall detection performance remains subpar, necessitating further optimization of the network structure. Xu et al. [10] proposed a detection method to address the issue of unpredictable target angles in UAV aerial images, aiming to generate prediction frames with more accurate information. Nonetheless, this approach entailed additional computational parameters and time-consuming computations due to the requirement of obtaining shooting angle information. Avola et al. [11] introduced the MS Faster R-CNN detection model, which effectively mitigated the issue of image quality degradation resulting from UAV motion shots. However, this model exhibits low detection speed. Zhu et al. [12] introduced the TPH-YOLOv5 model based on the transformer self-attention mechanism, utilizing the attention prediction head to replace the original YOLOv5 prediction head. This modification significantly enhances the target detection accuracy of aerial images, albeit at the cost of reduced detection speed. Du et al. [13] introduced a novel multi-target tracker, named GIAOTracker, that employs global information and optimization strategies. This method secured second place in the VisDrone 2021 MOT Challenge. The model comprises three phases: online tracking, global linking, and post-processing. The initial stage generates reliable trajectories based on camera motion, object motion, and object appearance information for each frame’s detection results. These trajectories are then refined using global cues and four post-processing methods. Habib Khan et al. [14] enhanced object localization and classification accuracy by introducing the decoupled detection head (DDH) in YOLOv4, enabling separate handling of classification and localization tasks. Huang et al. [15] introduced the BLUR-YOLO algorithm for target detection in UAV aerial images, which significantly enhanced both detection efficiency and accuracy.

To improve the aerial image detection ability under a complex environment with multiple small targets, mutual occlusion between targets, and limited computing resources, an aerial target detection algorithm based on improved YOLOv5 is proposed in this paper. Since the YOLOv5 model can reach 140 FPS, the fastest in image recognition, and can also guarantee better detection accuracy, we choose YOLOv5 as the benchmark model to improve the structure of this model to meet the detection requirements. Our proposed algorithm performs well and makes the following significant contributions in the domain.

- We propose an improved Mosaic algorithm to solve the redundant black-and-white boundary problem due to the different scales of the pictures.
- The hybrid domain attention module is added to the backbone network to improve the ability of feature extraction
- The feature fusion layer fused with P2 is added to the neck network, and the improved BiFPN network structure is adopted to improve the detection accuracy and network robustness.
- Finally, the VisDrone [16] dataset is used for detection, and it is experimentally proved that the improved algorithm improves the mAP by 6.1% compared with the original algorithm while meeting the real-time requirements.

## 2. The Principle of YOLOv5 Algorithm

**Figure 1:**
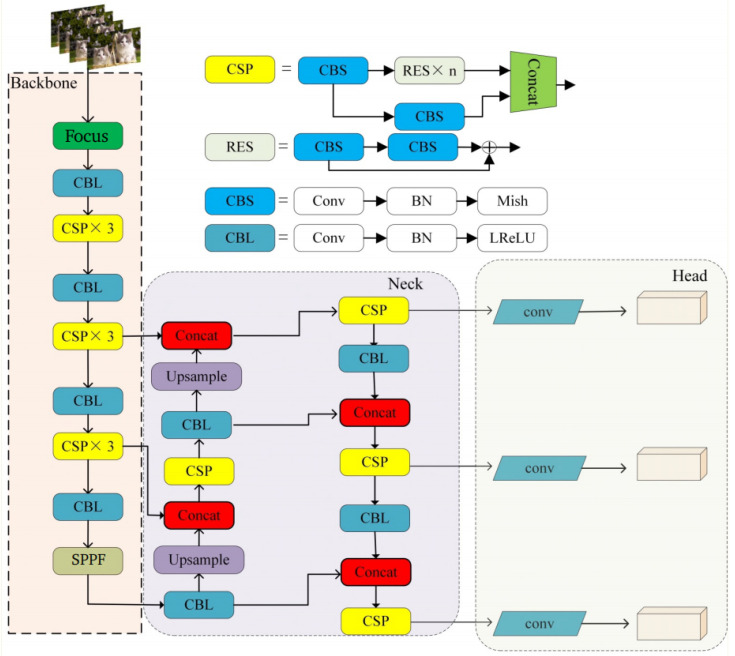
The network structure of YOLOv5.

The YOLOv5 [17,18] algorithm is a target detection algorithm that was open-sourced by Ultralytics in May 2020. Its network structure primarily comprises three components: the backbone network, the neck network, and the head network [19,20], as illustrated in Figure 1.

YOLOv5 [21]’s backbone network is built upon the CSPDarknet53 architecture. This incorporates a Focus network structure at the input end and an SPP network structure at the output end. The Focus network employs a slice operation with interval sampling to halve the size of the input image while quadrupling the number of channels, thus reducing the computational complexity of the model. Meanwhile, the SPP network effectively extracts rich feature information by utilizing four different-scale max-pooling layers in parallel to process the feature map. Activation functions employed in the model comprise the Mish function and the ReLU function, contributing to enhanced accuracy and stability.

YOLOv5’s neck network adopts the PANet + FPN network structure, facilitating the swift fusion of semantic information from the top layers with positional information from the bottom layers. This enhancement improves the localization information of deep features and enhances the detection accuracy of multi-scale targets in the model.

YOLOv5’s head network utilizes three different-scale detection heads: 19 × 19, 38 × 38, and 76 × 76, enabling the detection of small, medium, and large objects in the image. The CIoU [22] loss function is employed to mitigate issues related to non-overlapping bounding boxes. Finally, the optimal prediction boxes are selected using the non-maximum suppression (NMS) [23] technique.

## 3. Improvements to the YOLOv5 Algorithm

In response to the characteristics of small, densely packed, and occluded objects in aerial images, this paper proposes several enhancements to the YOLOv5 algorithm:

1. Data Preprocessing: The Mosaic algorithm is enhanced by aggregating nine images instead of four, thereby enhancing the model’s generalization and robustness.
2. Efficient Feature Extraction: The final CSP module of the backbone network is substituted with a hybrid-domain attention module to enable a more efficient extraction of meaningful feature information from the images.
3. Small Object Detection: To address the challenge of accurately detecting numerous small objects in aerial images, a prediction branch with a feature fusion layer incorporating P2 features is specifically added for small object detection.
4. Robustness Enhancement: To mitigate the loss of deep semantic information resulting from the incorporation of the small object detection layer, the FPN + PANet structure of the feature fusion network is eliminated. Instead, a simplified BiFPN [24] network structure is introduced to enhance the robustness of the network.

**Figure 2:**
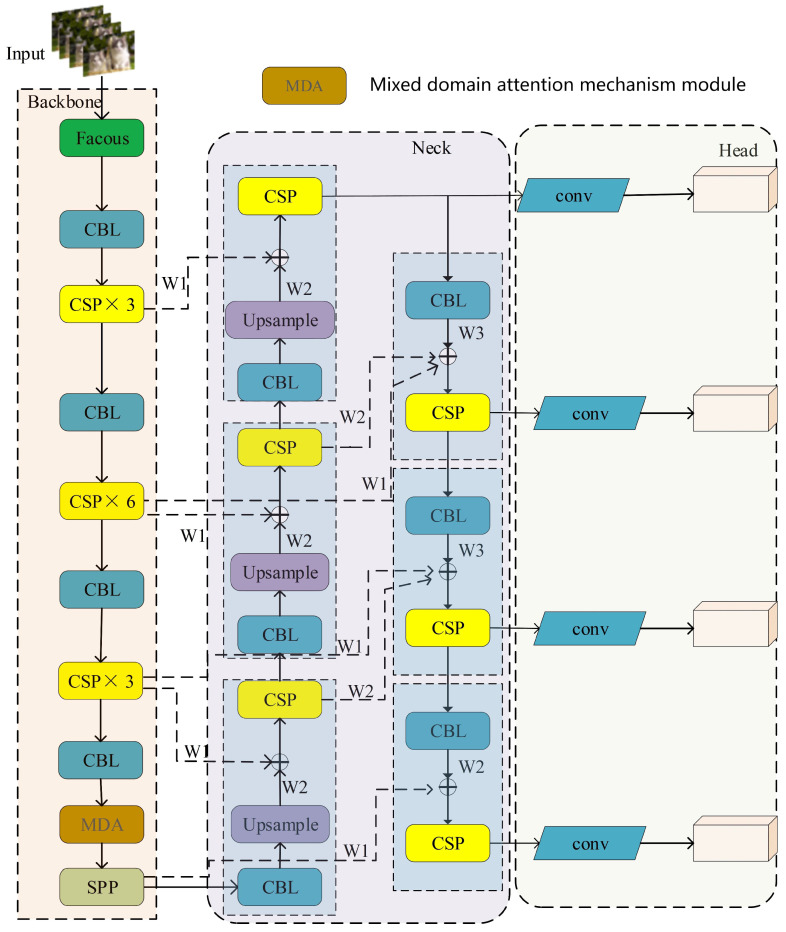
Improved YOLOv5 model.

The revised YOLOv5 algorithm’s network architecture is illustrated in Figure 2.

### 3.1. Improvements to Mosaic Algorithm

With the advancement of deep learning, standard data augmentation methods have become prevalent. However, researchers persist in exploring and refining data augmentation strategies. Based on the point of insertion of the augmentation step, these novel methods can be broadly classified into three categories: image transformation, image clipping, and image blending. Methods in the image transformation class typically involve random clipping after decoding the image, along with additional transformation operations. Notably, AutoAugment [25] and RandAugment [26] exemplify this category of methods, enhancing model performance through the automatic learning of optimal augmentation and generalization strategies. Conversely, methods in the image clipping category concentrate on clipping operations performed on rearranged images, often setting pixel values within the clipped region to 0 to achieve an expansion effect. CutOut [27] and RandErasing [28] stand out as representative algorithms in this category, enhancing the model’s generalization ability by randomly masking portions of the image. However, the first two categories of methods are constrained to transforming individual images, thereby limiting the extent of data diversity enhancement. In contrast, methods in the image blending category achieve a higher degree of data augmentation by blending multiple images to produce a new composite image. Notable algorithms in this category encompass CutMix [29], MixUp [30], and Mosaic, which hold greater promise for enhancing model accuracy owing to their capability to generate more diverse datasets. Given the efficacy of mix-and-match algorithms in data augmentation and generalization, this paper will center its attention on the Mosaic algorithm.

**Figure 3:**
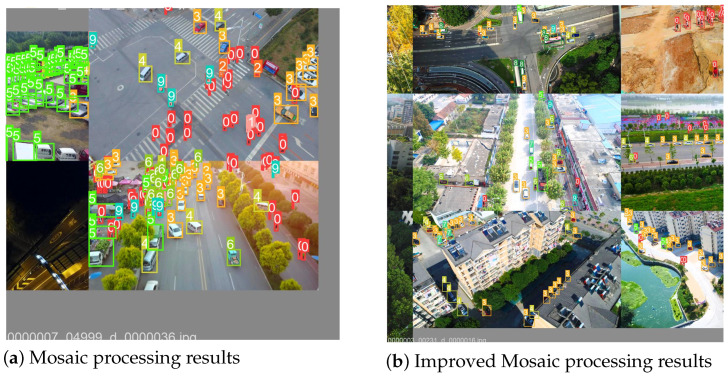
Example of Mosaic algorithm improvement.

The Mosaic algorithm is the default data augmentation method used in YOLOv5. This algorithm concatenates four images and feeds them into the model for training, which helps to increase the number of small objects, expands the diversity of objects in the dataset, and improves the model’s generalization ability. However, the original Mosaic algorithm randomly crops and resizes samples, which may result in cropping out all original objects, leaving only the background in the input sample. Additionally, due to the different scales of input images, many redundant black-and-white boundaries may appear in the concatenated image, extending the model’s training time. This situation is illustrated in Figure 3a.

To address these issues, this paper proposes improvements to the original Mosaic algorithm. First, the number of images concatenated is increased from four to nine. Then, the concatenated image is cropped based on the minimum area bounding box, followed by resizing. Compared to the random cropping of the original four images, this improvement minimizes the boundary area as much as possible, ensuring the integrity of objects. The processed images using the improved Mosaic algorithm are shown in Figure 3b.

The improved Mosaic algorithm significantly reduces the black-and-white boundaries, decreasing the proportion of irrelevant features in the images. Moreover, it dramatically increases the number of small objects in the dataset, leading to a better detection performance on small objects by the model.

**Figure 4:**
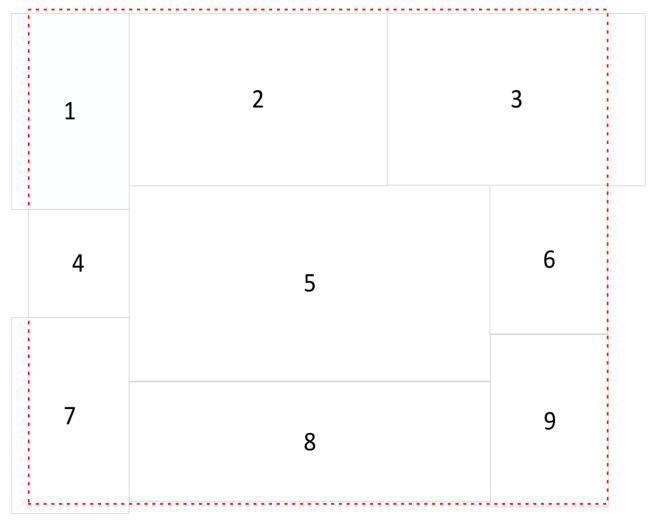
Minimum rectangular area.

The Mosaic algorithm enhances the quantity of small targets by merging four images, thereby augmenting the target count in the dataset and enhancing the model’s generalization ability. However, the original Mosaic algorithm randomly clips and scales samples, often resulting in the inadvertent clipping of all original targets, rendering the samples input to the model to consist solely of the background. Moreover, inconsistencies in the sizes of original images in the dataset, such as those in the VisDrone 2021 dataset, which mainly comprise images sized 960×540, 1920×1080, and 1500×2000, lead to the presence of numerous redundant black-and-white borders in images processed by the Mosaic algorithm. This results in the model obtaining a significant amount of redundant background information during the feature extraction process, thereby prolonging the model’s training time. Therefore, in this paper, we enhance the original Mosaic algorithm to address this issue. Initially, the number of splices is increased from four to nine, and the images are subsequently clipped based on the smallest area encompassed by the spliced images, as depicted in Figure 4, before finally being scaled. This improvement, compared to the original random cropping of four images, minimizes boundary areas and ensures target integrity. On one hand, the enhanced Mosaic algorithm significantly diminishes black-and-white boundaries, thereby reducing the proportion of irrelevant feature information in the image. On the other hand, it substantially augments the overall training data, concurrently enhancing the ratio of small targets in the dataset, leading to an improved detection performance on small targets by the model.

Increasing the number of spliced images from four to nine may affect the model’s ability to generalize across different aerial imagery scenarios, particularly the following:

1. Increased diversity: By splicing more images together, the model can expose itself to a wider range of background and contextual information during a single training session. This aids the model in acquiring a greater diversity of features, thereby enhancing its ability to generalize across various aerial image scenarios.
2. Enriching the target distribution: More spliced images result in more target instances and a more comprehensive target distribution. This assists the model in better understanding the characteristics of the target, such as shape, size, orientation, and occlusion, thereby enhancing detection performance.
3. Possible increase in noise: However, increasing the number of stitched images may also introduce more noise and interference. If there are significant disparities between the spliced images or if they are not spliced in a rational manner, it may cause the model to incorporate some extraneous noise features, potentially impeding its generalization ability.

This enhancement may influence the necessary training duration or computational resources, yet the impact can be mitigated through various measures, particularly the following:

1. Increased computation: Stitching together more images results in larger input sizes and more complex computational graphs. This results in a greater computational demand for each iteration, consequently prolonging the training duration.
2. Elevated memory demands: Larger input sizes and more complex computational graphs correspondingly escalate the memory requirements of the model. In the case of limited hardware resources, certain optimization measures may prove necessary, such as reducing the batch size or implementing more efficient computational strategies.
3. Requirement for hardware acceleration: To address this heightened demand for computation and memory, it may be necessary to utilize more potent GPUs or other hardware accelerators to expedite the training process.

### 3.2. Improvements to the Backbone Network

**Figure 5:**
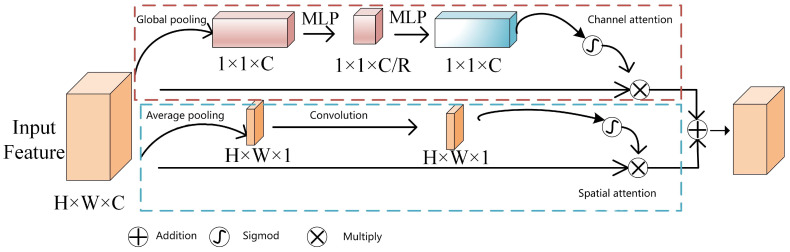
Mixed-domain attention mechanism module.

Considering the different focuses on the channel and spatial dimensions, where the channel dimension emphasizes what kind of feature information is meaningful while the spatial dimension focuses on where in the feature map the information is meaningful, considering only one dimension will inevitably lead to the loss of information in the other dimension. Therefore, this paper constructs an attention module based on the combination of channel and spatial dimensions. This module processes feature information in both dimensions in parallel. The specific network structure is illustrated in Figure 5. It is assumed that the feature map has dimensions W×H in spatial dimensions and C in channels. In the channel attention module, first, the feature passes through a global average pooling layer with dimensions W×H, resulting in a 1×1×C feature map. Then, this feature map is input into two multi-layer perceptrons (MLPs) for processing. In the first MLP, there are a total of *c*/*r* neurons, resulting in an output feature map with r channels. In the second MLP, there are a total of C neurons. Both use ReLU as the activation function. Finally, the resulting feature map is input into an activation function for processing. The entire process can be represented by Equation (1):

(1) $$S_{c}=\sigma \left(W_{1}\delta \left(W_{0}\left(F_{avg}^{c}\right)\right)\right)$$

where $F_{avg}^{c}$ represents the feature map after the output of the global pooling layer and $W_{0}$ represents the weight matrix of the first multi-layer perceptron, with dimensions of c×c/r, which is used to reduce the feature map from *c* dimensions to c/r dimensions. $W_{1}$ represents the weight matrix of the second multi-layer perceptron, with dimensions of (c/r)×c, which is utilized to restore the feature map from c/r dimensions back to *c* dimensions (the hyperparameter *r* denotes the dimensionality reduction factor), and *c* denotes the number of channels of the feature map. Finally, the obtained $S_{c}$ is multiplied with the input feature vector to obtain the output feature map of the channel attention mechanism. The channel attention mechanism is useful for obtaining information about what kind of features are useful from the global information but does not focus on the variation in spatial dimensions. Therefore, the feature maps are processed on the channel while adding an attention mechanism based on spatial dimension processing in parallel, which is performed as follows: firstly, the average pooling layer is used to transform the input features on the channel to obtain feature maps with dimensions H×W×1, which are then processed using the excitation function after passing through a single convolution operation, Then, after a convolution operation followed by activation function processing, the output feature map $S_{C}^{\prime }$ is obtained. The entire process can be represented by Equation (2):

(2) $$S_{C}^{\prime }=\sigma \left(f\left(F_{a\nu g}^{s}\right)\right)$$

where *f* denotes feature extraction using a 3 × 3 convolutional kernel, $F_{avg}^{c}$ denotes average pooling over the channel dimension, and the other parameters are the same as above. The resulting $S_{C}^{\prime }$ is then multiplied with the input features to obtain the spatially dimensioned feature maps (Ms). Finally, the input feature map after processing in both dimensions is summed to obtain the output map $I_{out}$ of the module, as shown in Equation (3).

(3) $$I_{out}=M_{c}+M_{s}$$

The hybrid attention module [31] consists of two components: the channel attention module and the spatial attention module. The former is tasked with learning the importance weights of each channel. Global statistical information for each channel is acquired by performing global average pooling or global maximum pooling operations on the input feature maps. Subsequently, this information is fed into either a fully connected layer or a convolutional layer to generate weight coefficients for each channel. These weight coefficients adjust the weights of the input feature maps in the channel dimensions. The spatial attention module is tasked with learning the importance weights of the feature maps in the spatial dimension. A spatial attention map is generated by performing a convolution operation on the input feature map. This attention map represents the importance weights of different spatial locations and is utilized to adjust the weights of the input feature maps in the spatial dimension.

The rationale behind the hybrid nature is that [32] channel attention and spatial attention can complement each other to improve the representation of the model. Channel attention focuses on identifying meaningful channels—those containing important information—while spatial attention focuses on identifying meaningful spatial locations. By integrating these two attention mechanisms, the hybrid attention module comprehensively captures key information in the image, thereby enhancing target detection accuracy. In this study, the hybrid attention module is incorporated into the final part of the backbone to enhance the model’s feature extraction capability. By adaptively learning the importance weights for each channel and spatial location, the model prioritizes important features during target detection, consequently enhancing detection accuracy and robustness.

Incorporating hybrid attention modules into the backbone network typically offers advantages in computational efficiency and effectiveness over traditional attention mechanisms. The following provides specific comparisons:

1. Computational efficiency:
  - Parallelized processing: Hybrid attention modules are typically designed to be lightweight in order to enhance model performance while imposing minimal computational overhead. Certain efficient implementations enable parallel computation of channel attention and spatial attention, thereby fully leveraging hardware resources and reducing computation time.
  - Optimization strategies: The integration of hybrid attention modules can further enhance computational efficiency through various optimization techniques, including depth-separable convolution, group convolution, or low-rank decomposition, aimed at reducing the number of parameters and computational complexity.
  - Dynamic adjustment: The hybrid attention module can also be dynamically adjusted based on the input feature map’s dynamic characteristics to avoid unnecessary computation. For instance, for simpler inputs, the model may require less attention computation to achieve optimal performance.
2. Validity:
  - Feature selection: The hybrid attention module effectively focuses on crucial features in both channel and spatial dimensions, enhancing the model’s ability to capture key information in complex scenes and improving target recognition accuracy.
  - Robustness Improvement: Through adaptive adjustment in feature map weights, the hybrid attention module enhances the model’s robustness to various noises and interferences, which is particularly crucial for target detection tasks in real-world applications [33].
  - Performance improvement: Experiments conducted on multiple benchmark datasets demonstrate that integrating the hybrid attention module into the backbone network typically leads to significant improvements in the performance of the target detection model, including metrics such as accuracy, recall, and mAP.

### 3.3. Neck Network with Fusion of P2 Features

**Figure 6:**
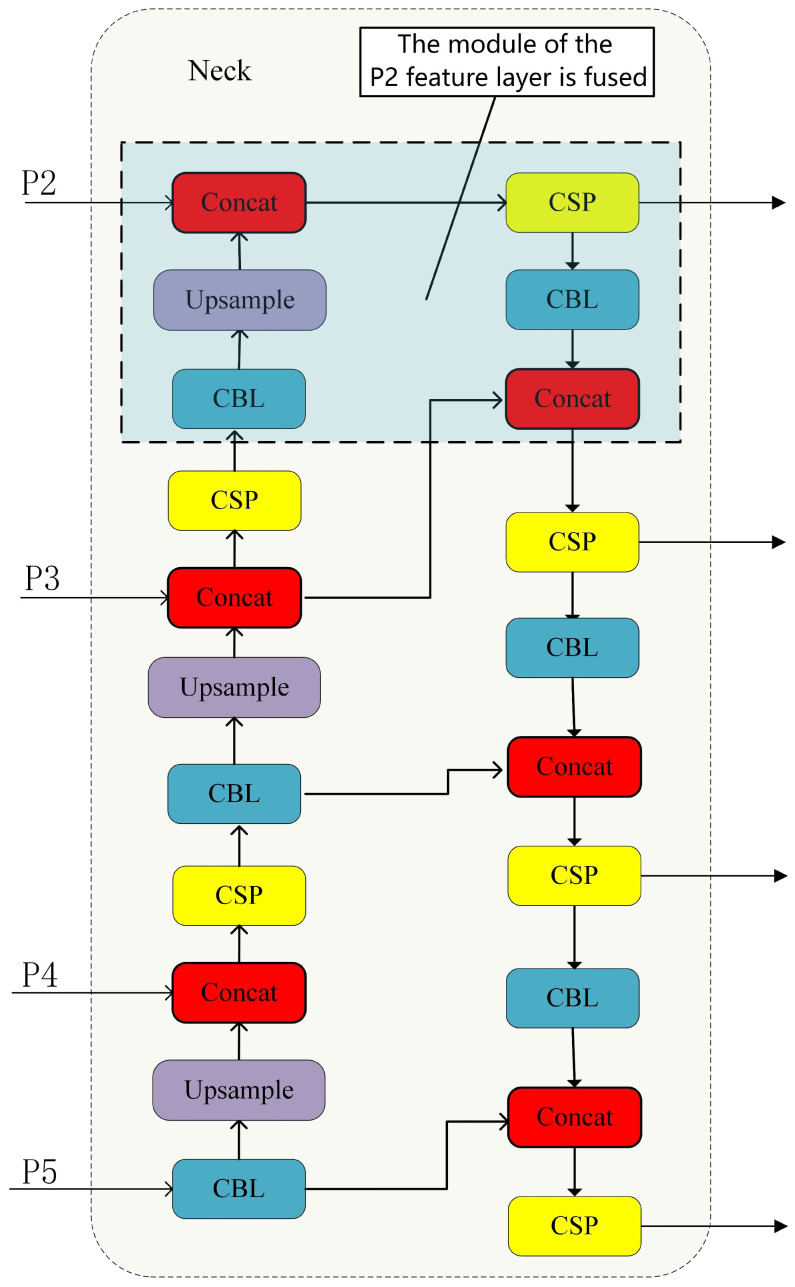
The addition of P2 feature fusion layer.

The neck network of the YOLOv5 algorithm primarily merges the last three feature layers from the backbone network. These three feature layers belong to the deep feature layers of the network. However, in deep convolutional neural networks, deep feature layers mainly contain semantic information of the input features. The semantic information extracted by the feature extraction network needs to go through downsampling from the previous layer. Each downsampling step leads to the loss of feature information. Therefore, deep feature layers are more suitable for detecting large objects. Since most objects in aerial images are small, and they occupy a small proportion of pixels, they inherently contain less feature information. Additionally, deep feature layers undergo multiple downsampling steps, leading to the loss of effective feature information for small objects multiple times, ultimately resulting in lower detection accuracy for small objects. Therefore, merely merging the features of layers P3, P4, and P5 cannot achieve the desired effect in small object detection. To further improve the detection accuracy of small objects, it is necessary to utilize the P2 feature layer, which has almost no loss of feature information. This enables it to fully participate in the fusion process with other feature layers. Hence, this paper adds a feature fusion layer and detection head to the neck network of YOLOv5, specifically designed for detecting small objects. The improved network model structure is illustrated in Figure 6.

Compared to the original network structure of YOLOv5, this model introduces an up-sampling process to the original neck network to incorporate feature information from the P2 layer [34], which enhances the representation of small targets. The entire fusion network comprises three up-sampling processes, with the P2 feature layer concatenated with the output of the third corresponding up-sampling to create a prediction branch with a size of 160×160. With an input image size of 640×640, this prediction branch can detect targets of 4×4, which is significantly more efficient and effective than the original YOLOv5 network, which can only detect targets of 8×8 or larger. By detecting targets as small as 4×4, it enhances the model’s ability to effectively detect very small targets and improve detection accuracy for small targets.

The benefits of this approach over other potential features or layers are specifically as follows:

1. Better small target detection: By fusing high-resolution features from the P2 layer, the model can better capture and detect small-sized objects in the image.
2. Multi-scale information fusion: Combining feature information from different scales can help the model to better deal with scale variations, thus improving detection performance.
3. Information richness: Fusing features from different layers can provide the model with richer semantic information and spatial details, which helps to improve detection accuracy and robustness.

### 3.4. BiFPN and Its Simplifications

**Figure 7:**
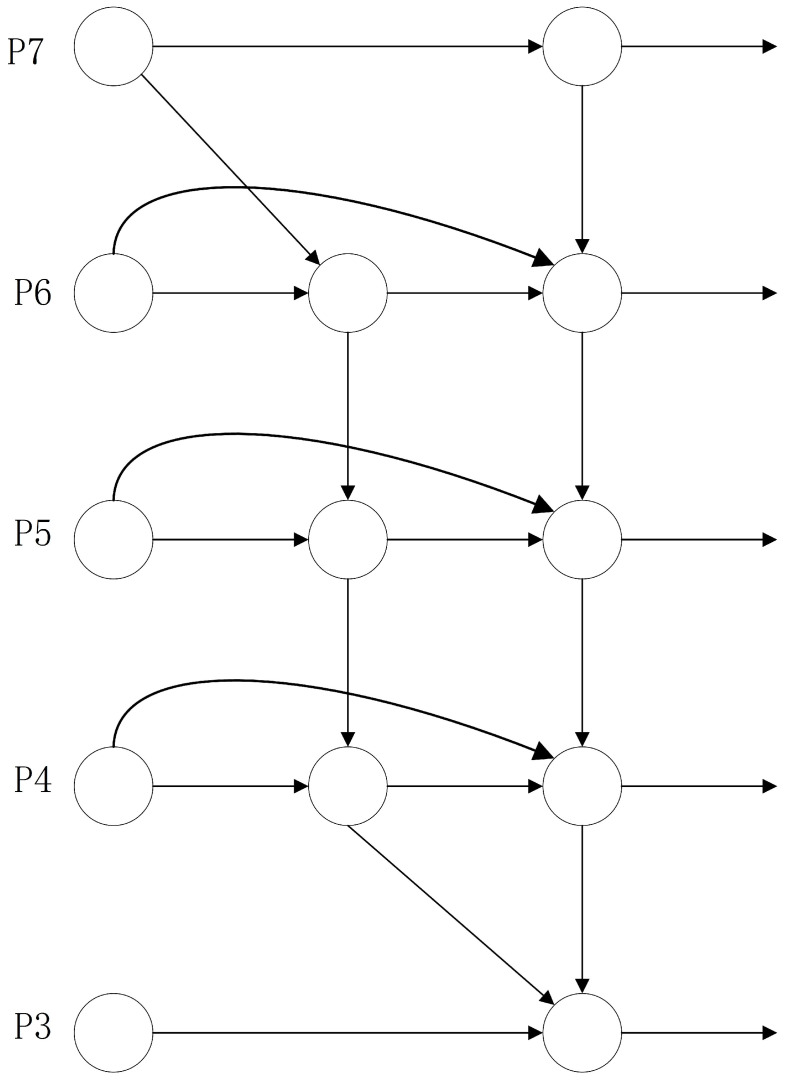
BiFPN network structure.

While adding a feature fusion layer incorporating the P2 feature can acquire more shallow features and enhance the detection accuracy of small objects in the neck network of YOLOv5, the PAN + FPN network structure of YOLOv5 introduces two additional branches, one from top to bottom and one from bottom to top, resulting in an excessive retention of shallow semantic information in the model and significant loss of deep feature information. Consequently, this paper removes the original feature fusion network and substitutes it with a bidirectional feature pyramid network (BiFPN) [35,36] to address this concern. The structure of the BiFPN network is illustrated in Figure 7 and it has the following three characteristics:

1. Elimination of unilateral inputs to reduce computational complexity.
2. Incorporation of residual connections for feature layers at the same scale to enhance feature representation capability.
3. Allocation of a weight to each feature layer involved in fusion, indicating their respective contributions to the fusion process.

**Figure 8:**
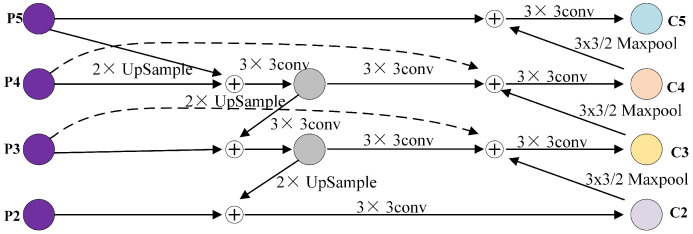
Improved BiFPN structure.

Since YOLOv5 originally consists of only five feature layers and the original network merges three feature layers (P3–P5), the addition of the P2 feature layer results in only four feature layers available for fusion. Consequently, corresponding improvements need to be made to the BiFPN. In this paper, one feature layer is removed from the BiFPN network, and the fusion is conducted on the P2–P5 feature layers. The improved network structure is illustrated in Figure 8. While this improvement may introduce a slight increase in computational complexity, the enhanced network model exhibits better detection performance for small and dense objects.

This paper adopts the optimized BiFPN to replace the PAN + FPN network structure, removing one feature layer of the BiFPN and opting to fuse the P2–P5 feature layers. Additionally, specific optimizations of the BiFPN include the following:

1. Cross-scale connectivity optimization: The BiFPN facilitates more comprehensive feature integration between different layers through top-down and bottom-up pathways, in contrast to traditional FPNs and PANs, which primarily employ top-down feature propagation. The optimized BiFPN enhances these cross-scale connections, thereby improving feature fusion efficiency.
2. Weighted feature fusion: BiFPN introduces learnable weights for each connected edge, enabling the model to dynamically adjust fusion based on different features. This weighted approach optimizes the fusion effect of multi-scale features, enhancing the accuracy of feature representation. In an optimized BiFPN, these weights may be fine-tuned using more refined training strategies or regularization methods.
3. Removal of redundant feature layers: Reducing the complexity and computational burden of the model can be achieved by eliminating one feature layer in the BiFPN and concentrating on fusing the P2–P5 feature layers. This decision preserves the most critical feature layers while eliminating those that may have less impact on performance.
4. Fusion strategy adjustment: When integrating the P2–P5 feature layers, various fusion strategies can be employed, including element-wise summation, element-wise product, or channel cascading. The optimized BiFPN may select the fusion strategy most suitable for the task to maximize the impact of feature fusion.

The impact of this method on retaining semantic information is that, with enhanced cross-scale connectivity and weighted feature fusion, the optimized BiFPN effectively conveys and preserves deep semantic information [37]. This facilitates a more accurate identification and localization of targets in detection tasks by the model. Focusing on fusing the P2–P5 feature layers ensures that the model possesses an adequate feature representation capability for addressing targets at varying scales while mitigating the interference of redundant information.

The impact on model size and inference speed is that the removal of redundant feature layers can decrease the number of parameters and computational complexity of the model, thereby reducing both model size and inference time. This is particularly crucial for resource-constrained application scenarios. Nonetheless, weighted feature fusion and enhanced cross-scale connectivity may impose some additional computational burden.

## 4. Experiments and Results Analysis

### 4.1. UAV Aerial Dataset

To assess the effectiveness of the improved model, this paper utilizes the VisDrone aerial dataset for training and testing purposes. The images in this dataset are primarily captured from various cities, covering diverse and complex environments, thereby offering a stringent evaluation of model performance. Moreover, to further challenge the performance of network models, this dataset includes a challenge set comprising 1580 images. Small objects comprise three-quarters of the entire dataset, with each category exhibiting a certain degree of occlusion.

### 4.2. Experimental Environment and Training Methods

The experimental environment in this paper is configured on a server station, featuring an Intel(R) E5-2687W V4 CPU @3.0 GHz (Intel; Mountain View, CA, USA), and an NVIDIA GeForce RTX 3090 GPU (with 24 GB of VRAM) (NVIDIA; Santa Clara, CA, USA), supported by 24 GB of RAM and a 1TB hard drive. The operating system employed is Ubuntu 18.04 LTS (Canonical Ltd.; London, UK), equipped with CUDA version 11.0. The deep learning framework utilized is PyTorch, with Python version 3.8.

To fully leverage the effectiveness of pre-trained weights in the improved YOLOv5 algorithm, this paper adopts a technique involving freezing and unfreezing to enhance training efficiency. During the freezing phase, the feature extraction network is frozen, and the weights in this part remain unchanged, resulting in minimal resource consumption. In the unfreezing training phase, the weights in the backbone network are adjusted, necessitating more computational resources. Therefore, the batch size is set to 8, the learning rate to 0.0004, the momentum factor to 0.937, and the input image resolution to 1152×1152.

### 4.3. Ablation Experiments

**Figure 9:**
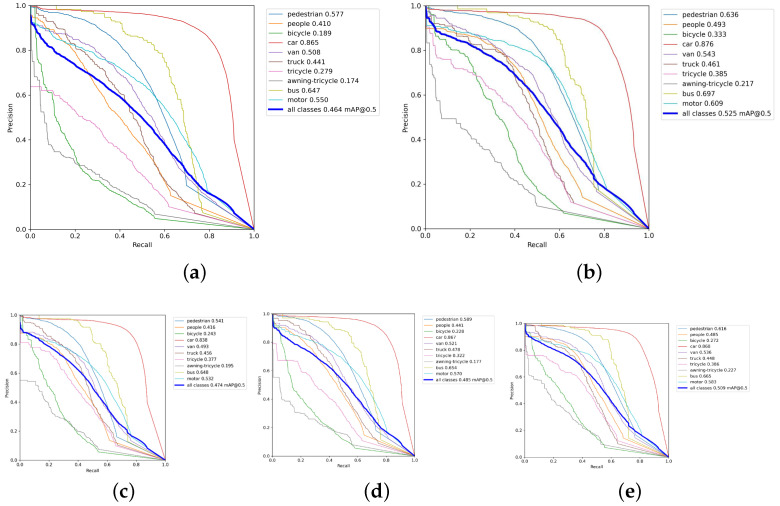
mAP values of various types before and after improvement. (**a**) Varying detection accuracies observed in YOLOv5l. (**b**) Varying detection accuracies of the improved model. (**c**) Detection accuracies of various categories for the improved model of Mosaic’s algorithm. (**d**) Detection accuracies for various categories of the improved model of the backbone network. (**e**) Detection accuracies of various types of enhanced and refined models operating within the designated P2 layer.

To validate the effectiveness of multiple improvements, this paper conducted ablation experiments on the VisDrone aerial test set. YOLOv5l release V6.0 was used as the baseline model for comparison, with mAP(0.5), GFLOPs, FPS, and model parameter count as evaluation metrics. The results of the ablation experiments are presented in Table 1, and the various mAP values for YOLOv5l and the improved model are illustrated in Figure 9.

Table 1 shows that the YOLOv5l model has 46.56 M parameters and 109.3 GFLOPs, with an average detection accuracy of only 46.4%, suggesting low detection accuracy. As shown in Figure 9c, post improvement with the Mosaic algorithm, the model size and floating-point operations remain unchanged, while the accuracy increases by 1%. This validates that the improvement enhances detection quantity, thereby improving the model’s detection accuracy.

As shown in Figure 9d, replacing the backbone network with a mixed attention module and SPPF module results in a 1.1% increase in accuracy, albeit with a minor sacrifice in floating-point operations and model size. As shown in Figure 9e, by incorporating the strategy of adding the P2 feature layer in the neck network, despite a certain degree of decrease in the model’s detection speed, the average detection accuracy increases by 2.4% to 50.9%, representing the largest improvement among all enhancements. This demonstrates that the addition of the P2 feature fusion layer effectively enhances the detection of small targets.

Lastly, with the introduction of the optimized BiFPN structure, the model achieves an mAP of 52.5% and a detection speed of 80.12 frames/s, satisfying real-time detection requirements. Figure 9a,b illustrate that the improved model demonstrates significant enhancements in various metrics compared to the baseline model. In particular, the bicycle and tricycle dense small targets exhibit the most substantial improvements, with increases of 14.4% and 10.6%, respectively, highlighting the improved model’s efficacy in detecting dense small targets in aerial images.

### 4.4. Comparative Experiments

To assess the superiority of the improved YOLOv5 over other aerial detection algorithms, this study conducted comparative experiments between the improved YOLOv5 algorithm and several mainstream aerial algorithms using the test set of the VisDrone dataset. The comparison results are presented in Table 2.

As indicated in the table, at an IoU of 0.5, the improved YOLOv5 algorithm surpasses DMNet, YOLOv3, Cascade-RCNN, and YOLOv5l by 4.9%, 15.9%, 6.6%, and 6.1%, respectively.

Considering Table 2, the ClusDet and PRNet models attain higher mAP values at a 0.5 IoU threshold. This primarily stems from the adoption of a three-stage overall structure by the ClusDet model, which affords a broader receptive field during feature extraction compared to the network structure employed in this study. Moreover, ClusDet employs higher-resolution input images compared to the 1152 resolution utilized in this paper. Consequently, for very small objects, the ClusDet network extracts more feature information, resulting in a higher mAP when the IoU threshold is set to 0.5.

However, at higher IoU thresholds, precision requirements for detection increase, and the significance of detecting very small objects diminishes. Therefore, in terms of mAP(0.5:0.95), the model proposed in this paper achieves higher detection accuracy.

Conversely, the PRNet model primarily utilizes a detection approach that integrates difficult and overall regions. Likewise, because of the relatively limited extractable features for very small target objects, as the IoU threshold increases, the PRNet network may struggle to accurately detect extremely small targets, leading to a comparatively lower mAP (0.5:0.95) value.

Ultimately, considering the comprehensive results from Table 2, even though the mAP may not attain its peak value when the IoU threshold range is set to 0.5, it remains comparable to that of ClusNet and PRNet. Nevertheless, with the threshold set to 0.5:0.95, the mAP value of the improved YOLOv5 algorithm achieves 34.0%, surpassing all other models.

### 4.5. Demonstration of Experimental Results

**Figure 10:**
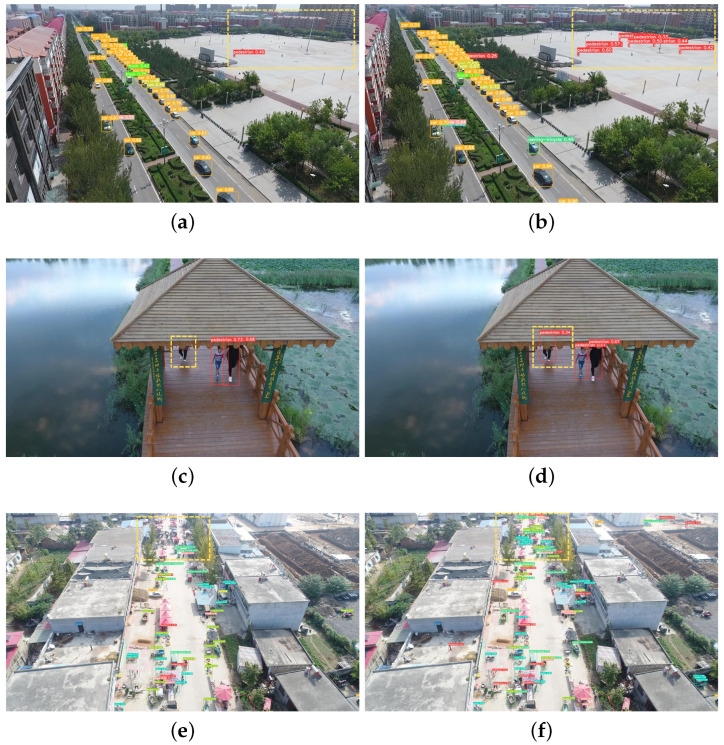
Detection effects in various scenarios. (**a**) Small target scene detection before improvement. (**b**) Small target scene detection after improvement. (**c**) Occlusion scene detection before improvement. (**d**) Occlusion scene detection after improvement. (**e**) Dense scene detection before improvement. (**f**) Dense scene detection after improvement.

To validate the detection performance of the improved YOLOv5 algorithm in real-world scenarios, we conducted tests on the challenging set of the VisDrone aerial dataset, which comprises the most complex scenes with the highest detection difficulty. Partial detection results in various scenarios are depicted in Figure 10. Observing the images, it becomes evident that, in scenarios with small targets, severe occlusion, or dense scenes, the improved algorithm demonstrates higher detection accuracy, stronger robustness, and lower false detection rates, thereby showcasing superior detection performance.

## 5. Conclusions and Future Work

### 5.1. Conclusions

This paper proposes an improved YOLOv5 algorithm for aerial image object detection, targeting the complex backgrounds, dense distribution of small objects, and overlapping instances present in aerial datasets. In the data preprocessing stage, the Mosaic algorithm is enhanced to increase the number of training targets, thereby improving the model’s detection accuracy. To effectively extract useful feature information from input images, a hybrid attention module is added to the backbone network. To enhance the detection accuracy of small objects, a strategy of adding a fused P2 feature layer to the model’s neck network is employed. Additionally, to better integrate shallow and deep features, the PAN + FPN network is replaced with the optimized BiFPN network. Experimental results demonstrate that the improved model achieves a 6.1% increase in detection accuracy compared to the baseline model and meets real-time detection requirements. Furthermore, when compared with advanced aerial image object detection algorithms, the proposed model achieves the best overall detection performance in terms of average detection accuracy.

### 5.2. Future Work

Comparing the improved YOLOv5 with state-of-the-art aerial image detection algorithms, such as the recent research from Zhejiang University—combining the real-time detection and recognition (RT-DETR-X) model with the SAHI (slice-assisted hyper-inference) method [44] for detection using the VisDrone-DET dataset—the improved YOLOv5 slightly lags behind the RT-DETR-X model, but YOLOv5 has superior processing speed and a higher FPS rate, which is crucial for real-time surveillance applications.

In this paper, YOLOv5 has been improved and optimized in many aspects to improve the detection effect of the model in the task of aerial image target detection, but there are still many problems to be improved. Regarding the improvement of the Mosaic data augmentation algorithm, although the overall data are expanded, the original data have fewer categories, where, after the improvement, there is still not much change in the proportion of the overall number of occupants; only their total number increases. How to perform data augmentation for the lower number of categories without increasing the number of other categories in order to reduce the training time is the focus of the next research.

With the continuous evolution of UAV technology, it is expected that more researchers will be attracted to the construction of aerial photography datasets in the future in order to gradually fill the gaps in existing UAV datasets. However, there are significant differences among UAV datasets due to the diversity of UAV models, collection regions, and shooting targets. Therefore, how to effectively utilize different datasets for pre-training to obtain the relevant pre-training weights and then improve the detection accuracy of the model for certain categories on specific datasets will become the core issue of the subsequent research. In short, future research will focus on pre-training methods across datasets with a view to optimizing the detection performance of the model.

## Figures and Tables

**Table 1:** Ablation experiment table.

Model	mAP (0.5)	GFLOPs	FPS (Frame/s)	Quantity of Participants (M)
YOLOv5l	46.4%	109.3	89.65	46.56
Mosaic improvements	47.4%	109.3	89.34	46.56
Backbone network improvements	48.5%	109.5	88.12	46.69
Addition of P2 layer	50.9%	140.5	82.33	52.76
Fusion BiFPN	52.5%	152.1	80.12	54.77

**Table 2:** Comparison of different algorithms on the VisDrone dataset.

Arithmetic	mAP (0.5)	mAP (0.5:0.95)
DMNet [38]	47.6%	28.2%
YOLOv3 [39]	36.6%	17.5%
Cascade-RCNN [40]	45.9%	24.3%
YOLOv5l	46.4%	28.1%
QueryDet [41]	48.2%	28.3%
ClusDet [42]	53.2%	30.4%
PRNet [43]	53.9%	32.0%
Model of this paper	52.5%	34.0%

## Data Availability

Data are contained within the article.
